# Introducing Progressive Strength Training Program in Singapore's Elder Care Settings

**DOI:** 10.3389/fmed.2021.515898

**Published:** 2021-09-30

**Authors:** Magnus Björkgren, Frank Borg, Ken Tan, Gerd Laxåback, Lisette Nygård

**Affiliations:** ^1^Health Science Unit, Kokkola University Consortium Chydenius, Kokkola, Finland; ^2^Pulsesync Pte Ltd., Singapore, Singapore

**Keywords:** strength training, exercise as medicine, elder care, implementation, interRAI

## Abstract

The use of progressive strength training among the elderly has become an accepted part of evidence-based practice for preventive and rehabilitative care. Exercise is undoubtedly one of the pillars for resilient aging. While research has shown impressive outcomes from strength training, the challenge remains to get elderly persons to exercise. Here we describe a Finnish-Singaporean cross-national project that provides a unique opportunity to evaluate the implementation of strength training in settings where it had previously not been applied. We report from the first 2 years of implementation using assessment data and surveys directed to frontline therapists responsible for the implementation. The strength training concept was progressively implemented in 24 elder care locations in Singapore including residential homes, day rehab/care centers, and senior activity centers. Each location was provided with training, support, gym equipment and technology solutions. It remained for individual sites to enroll elderly to the program, to perform assessments, and to direct the progressive strength training. Based on data from the first 2 years of implementation, improvements in lower body muscle strength were found in Leg Curl (ave 11.1–48.8%), Leg Extension (ave 10.2–24.0%) and Hip Abduction/Hip Adduction (ave 7.0–15.8%). Of the trained therapists, 95% strongly agreed or agreed to some extent that the implementation had been successful. The practice-based evidence from the project has demonstrated that it is feasible to implement progressive strength training in real life settings, using technology. While the implementation initially required handholding and support, the approach yielded consistent improvement rates in muscle strength comparable to results from randomized clinical trials (meta-analysis studies). Significant improvement rates in muscle strength were found in all three types of sites, demonstrating that gym training can be employed broadly in elder care. The Senior Activity Centers offer an interesting model for reaching seniors with preventive actions at an early stage. The data support a 3-month training as an effective intervention of introducing strength training in elder care settings, promoting healthy aging.

## Introduction

The aging population presents a global challenge. Old age is often associated with limitations of physical function, frailty, chronic diseases, and a consequent increase in the need for health services which translate into a higher economic burden for the society. The decline in physical functions and onset of chronic diseases also affect the elderly's quality of life. This has triggered a societal investigation in attenuating the age-related decline in physical function while increasing the years on independent living. A growing body of systematic evidence supports the importance of both exercise therapy and physical activity as prevention and treatment of specific diseases (1, 2) and for promoting healthy aging (3–8). Supervised intensive training has, according to the studies, clinically significant effects on strength and physical function (9–13). Global initiatives such as “Exercise is medicine” (14) demonstrates the awareness of the importance of exercise as a complement of the clinical treatments. Muscle strength can be improved regardless of age making it a broadly applicable remedy. The Finnish current national care guidelines have adopted “exercise is medicine” for an array of diseases and conditions (15). The Finnish FINGER and DR's EXTRA studies (16, 17) have highlighted physical activity and exercise as preventive factors against dementia. In the UK, The Academy of Medical Royal Colleges has in their 2015 guidelines referred to exercise as the “miracle cure” (18). The Lancet (19) initiated in 2012 its Series on physical activity and concluded (20) “that physical inactivity is as important a modifiable risk factor for chronic diseases as obesity and tobacco.” WHO (21) has addressed the issue of physical (in)activity on a global level. WHO (22) has also recently introduced the concept of intrinsic capacity (IC) to emphasize the potential of healthy aging and combatting frailty though modifiable factors such as physical activity.

Progressive strength training in particular, is an important exercise therapy (23), indeed (24) “resistance exercise training should be considered a first-line treatment strategy for managing and preventing both sarcopenia and dynapenia.” Since the 1990s, studies have shown the feasibility and effectiveness of progressive strength training for the elderly (25–28). Progressive strength training has recently (29) been emphasized by The Asia-Pacific Clinical Practice Guidelines for the Management of Frailty as a top priority method to combat frailty: “We strongly recommend that older adults with frailty be referred to a progressive, individualized physical activity program that contains a resistance training component.” This echoes earlier reviews (30). Furthermore, there is also molecular evidence of the benefits of exercise (31).

While evidence-based research and practice have shown the importance of exercise and physical activity for health, the challenge remains to get people to exercise and to introduce exercise in clinical and other settings of elder care. There are many excuses not to exercise, but aging should not be one of them. The question is how to adapt academic research on evidence-based practices (EBP) to concrete situations “outside the laboratory” and embed them in the real world. This touches on the topic of “practice based evidence” (PBE) which is about developing evidence from real-life practices (32–34). Despite vast scientific knowledge, there are seemingly challenges and bottlenecks in moving from trial to practice. The barriers could be financial, technological, educational but also a shortage of therapists and trainers. Through a series of implementation projects in Finland, we have found that the availability of technology itself is often not enough. In most cases, there is a need for facilitators and a need to integrate multiple solutions into workable packages.

In the area of strength training Singapore's elder care has provided a unique opportunity for gathering practice-based evidence. Gyms for the elderly has not been part of standard practice, that is, none of the participating sites had previously applied gym technology for strength training. To facilitate the implementation three companies and a University partner came together to develop a solution. The package presents a combination of gym technology and assessment instruments making it possible to evaluate the persons' physical condition and training on a continuous basis. The project, Gym Tonic (35), was funded by the Lien Foundation to support the implementation. Using the PBE perspective we report on the results from the first 2 years of running the program. The key study questions include: (i) what we can learn from real-life practice patterns in introducing strength training for elderly, and (ii) how the results compare to studies performed in more controlled environments. The study object provided a unique opportunity to investigate the start-up phase of strength training in settings where these concepts had previously not been applied. This is the principal aim of the study. The conclusions are limited to this phase of adopting technology for strength training. Only limited data was available for longer follow-up periods.

## Methods

Each site was provided with a selected set of gym training machines (for core muscle group training), standardized assessments and targeted training for the therapists and specialists running the program. The adoption of new technology was planned as an implementation project. The companies provided the technological base for Gym Tonic (measurement devices, gym machines, and IT-solutions), and developed an integrated software solution for pulling different information systems together. The University partner played the role of research investigator, designing assessment protocols, training therapists and specialists from the participating organizations. To entice the elderly to first join and then stay on the Gym Tonic program, a behavioral change strategy was adopted, primarily making the exercise regime safe, simple, hassle-free and motivating.

Altogether the Gym Tonic concept comprises five key components:

- **Gym technology**: Six pneumatic machines which focus on the core muscle groups, which are safe (exercises in sitting position), and gentle on the joints.
- **Assessment technology**: standardized assessment instruments for measuring pre and post training status.
- **Training of therapists/specialists**: uniform training/ education program for therapists and specialists from participating organizations.
- **Training intervention**: recommended progressive strength training periods, that is, 2 times a week for 12 weeks.
- **Behavioral change strategy**: allowing the elderly to exercise in everyday clothes, keeping training simple and short, pre-programmed exercises using RFID smart card to activate each machine.

### Gym Technology

The gym technology used for the strength training includes the following machines, Leg Extension/Curl, Leg Press, Chest Press, Lat Pull, Abdomen/Back, and Hip Abduction/Adduction (36). The exercise equipment is based on air pressure technology making exercise safer for the elderly. The exercise equipment is designed to match the body's natural muscular movement. The software attached to the machines records all activity for automatic reporting. Individual exercise levels can be programmed into the system with a SmartCard/Touch technology. The system has also a built-in progressive resistance algorithm: whenever the person performs an excess two or more repetitions than programmed for during a session, the load will automatically increase the next session. The performance of each training session is saved in the training database. The Gym Tonic sites were encouraged to set up the gyms in “pleasant” environments to make the gym experience a positive one for the users.

### Assessment Technology

The protocols for assessing physical functions were designed using existing and validated tests. The objective was to apply measurement technology whenever possible, thus giving the most accurate measures of physical functions. The assessment protocol (Welmed) included isometric strength measurements with Leg Extension/Curl and Hip Abduction/Adduction. The isometric strength measurements were conducted by attaching a Performance Recorder unit to the gym devices (36). All strength measurements have three performances with the best performance being recorded as the result. The result is measured in nominal units of kilogram force (kgf) but can also be converted to torque units Newton-meter (Nm). For most tests, the strength measurements are normalized by dividing the strength results by body mass (kgf/kg or Nm/kg). The assessment protocol also included grip strength and body composition (37), balance test [Hur force platform BT4[36]], functional tests, for example, five times sit to stand (FTSTS), Berg Balance Scale (BBS) (38), and 4 m timed walk. Information on functional dependency including Activities of Daily Living (ADLs), cognitive functions, and communication was provided by the interRAI assessment (39). Together, the assessments served as a basis for setting personal goals for the training period and to evaluate the effectiveness of the resistance training.

### Training of Therapists/Specialists

The training of clinical staff was conducted in Finland. Two persons from each site, mainly physiotherapists and exercise therapists, were sent to Finland to be trained. The 4-day course included performing the assessments, how to conduct progressive strength training with elderly, how to create an individual exercise plan, and lectures on exercise as medicine. In addition, 2 days were allocated for site visits to demonstrate how gyms for elderly are being operated in Finland. Four batches of frontline staff were trained in Finland during the first 2 years of implementation. After the training, the students had to perform five Welmed assessments in their own facilities cases prior to a written and practical exam (with a real participant) to be certified for Gym Tonic. The practical exams were carried out in Singapore, also giving the Finnish educators a possibility to audit the gyms and test sites to ensure data quality. The persons trained were given time to learn in practice before the final exam. Additional support was provided through five webinars during the 2 years of implementation. Some support was also provided by the local vendor in Singapore.

### Exercise Intervention

The recommended progressive strength training period for elderly was set at 3 months, twice a week with two sets of exercises at each machine. The average time for every session was around 30–45 min. The assessments were performed at the start and at the end of the training period, and follow-ups at 3-month intervals if the training continued. Printed participant profiles and progress reports were shared with the elderly when viable. The profile reports included target values developed for motivating the elderly to exercise. The progression of the training was suggested to be adjusted in terms of the repetition maximum (RM). The notation × RM means the resistance level at which one can do a given maximum number × of repetitions. A smaller number of maximum repetitions × mean s a higher resistance level. Resistance level had been set to 15RM for weeks 1–2, 10RM for weeks 3–7 and 8RM for weeks 8–12. Thus, with increasing load the maximum repetitions should go down. The sites were given freedom to roll out the implementation as they found best. It remained up to the Gym Tonic sites to enroll participants into the gyms, no rigid inclusion or exclusion criteria were used, as most people who have the capacity to function in a gym will benefit from strength training. The centers generally advised the elderly to seek doctor's clearance especially for those who had underlying medical conditions. It was up to the trained staff to perform the pre and post assessments, and to direct the progressive resistance training in the gyms with the help of assistants and other staff.

### Behavioral Change Strategy

To entice the elderly to join (or even try) and stay on the program, we adopted a behavioral change strategy primarily to make the exercise regime safe, simple, hassle-free and motivating. Pre-training briefing was conducted, and the benefits of strength training was shared with them. To make it hassle-free, the elderly were welcome to exercise in their everyday clothes. Their pre-programmed exercises were automatically retrieved from their RFID smart card and there was no need for them to remember or manually adjust any loads. To make it simple and easy, training intervention was also kept short, around 30–45 min, and the elderly were only expected to exercise twice a week. During the training period, the therapists and specialists were encouraged to update the elderly on their progress.

### PBE Approach

The sites were given considerable freedom to roll out the implementation. This was deliberate as we wanted to evaluate the adoption of strength training in real environments. The main goal of the research was to learn from the implementation using the data, subsequently randomization, control groups etc. was not part of the set up. It was up to the sites to recruit participants to the program, based on who they thought could participate in gym training. Participation was voluntary and safety considerations were assessed by the health professionals at the sites. From clinical trials, we already know that strength training is very effective for the elderly. The PBE approach means taking what is known for from clinical trials, putting knowledge into practice, and evaluating the results. The main issue is then whether the adoption of the concept has resulted in successful outcomes or not. This can be evaluated mainly by comparing the results from practice to results research (meta studies) in the field. In addition, we used reference groups in Finland and Singapore where we had applied identical technology for both strength training and follow up. Finally, we targeted the frontline therapists with questionnaires to gather data on the experiences from the implementation.

## Results

We limit the discussion to the adoption of strength training and measurable outcomes mainly strength improvements.

### Participant Characteristics

A total of 24 locations in Singapore had adopted the Gym Tonic approach at the time of data analysis. Assessment labs/gyms had been successfully set up in three types of settings:

- Residential Facilities (7 sites)—RES
- Day Rehab/Dementia Day Care/Senior Care Centers (14 sites)—DAY
- Senior Activity/Community Centers (3 sites)—ACT.

**Figure 1:**
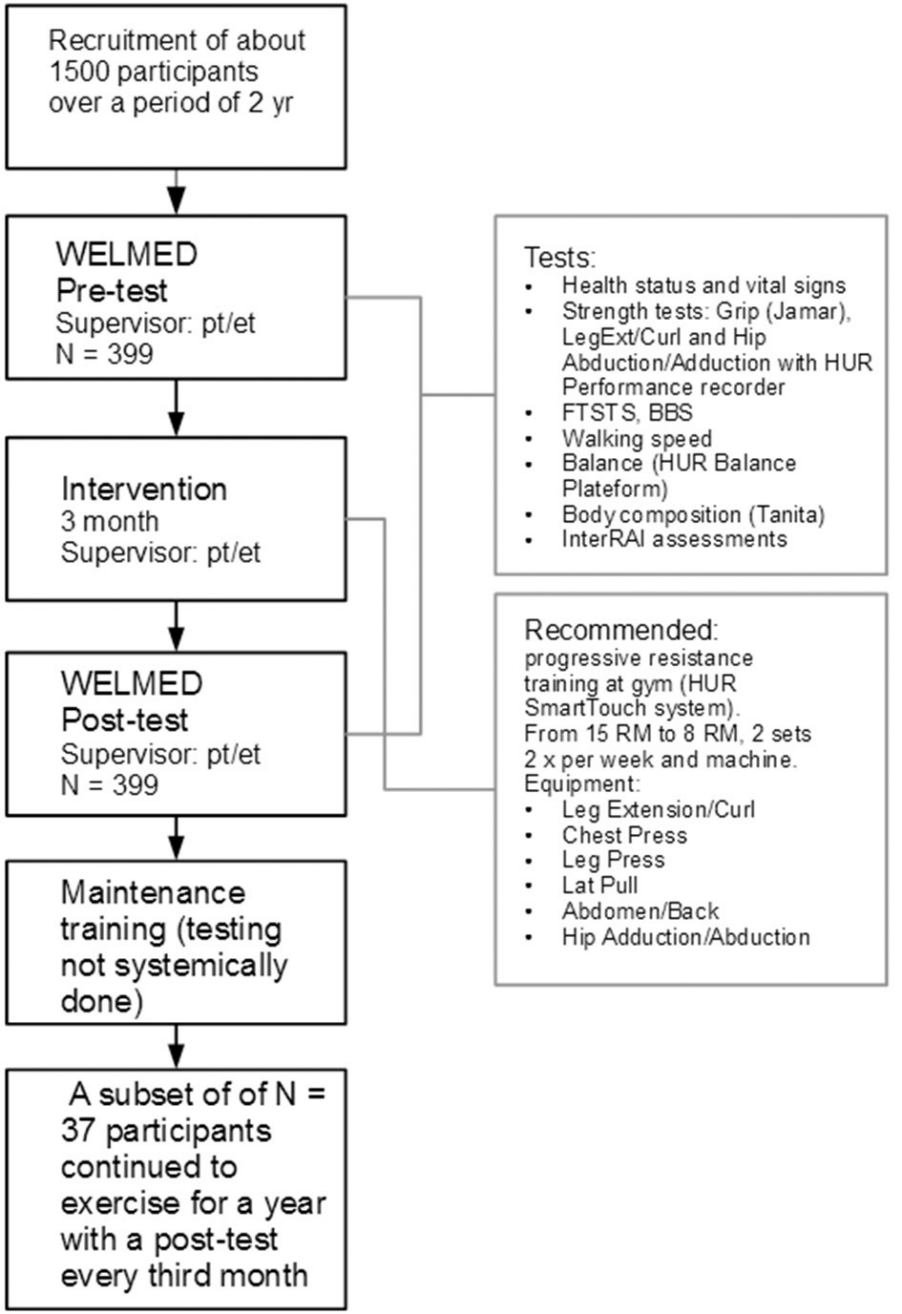
The Gym Tonic process and the data collection. Thousand five hundred participants were registered. The sites were asked to deliver data on those who had properly completed the 12-week training and the pre and post assessments (*N* = 399). Welmed stands for a test protocol summerized by the upper right box. FTSTS, five times sit to stand; BBS, Berg balance scale; pt, physiotherapist; et, exercise therapist.

These were the number of sites that had received funding by the Lien Foundation for setting up the gyms. The pooled data obtained from the first 2 years of implementation provided the research data. Measurement and assessment data from 399 persons (72 ± 11.4 y, M 53%, F 47%) were included in the research database. These persons were checked and confirmed to have participated in at least one 3-month gym intervention period. During the implementation period about 1,500 participants had registered for Gym Tonic (Figure 1). All who entered the training program were not assessed. In a real-life implementation there is naturally a learning period and not enough resources to assess everybody from the start. This explains the lower number in the data set available for analysis. For the analysis, the sites were asked to deliver data on those who had properly completed the 12-week training and the pre and post Welmed assessments (*N* = 399). Looking at outcomes mainly in strength this selection bias is necessarily not a problem as most people are regarded to benefit from strength training. This specific data set gives a profile of the clients recruited into the gym training groups, how the participants trained, and the effectiveness of the training in terms of improvement rates. The dropout rate from the training was about 10% mostly due to factors like hospitalization, acute events, death, house moving, and some for lack of motivation. A second smaller data subset included elderly who had completed 1 year of intervention and had also completed post assessments quarterly for us to analyze. While most people continued with one training session per week, not many organizations completed post-assessments diligently after the 3-month intervention. Most sites focused only on assessing the initial 12-week training period. Hence we had only 37 residential participants in the 1 year follow-up group.

Table 1 shows the basic characteristic of the participants using three interRAI scales; ADLh (Activities of Daily Living Hierarchical Scale, 0–6), CPS (Cognitive Performance Scale, 0–6), and COMM (Communication Scale, 0–8). These scales measure the performance status from independent to totally dependent. Most of the participants in the data set were relatively independent in ADLs which is a measure of physical functions. About 85% of the persons were independent or needing only supervision (ADLh = 0,1). More differences were seen in cognitive functions. The average cognitive performance in the RES groups was at the mild impairment level (CPS = 2.01), in the DAY group slightly over borderline intact level (CPS = 1.29), and for ACT close to intact (CPS = 0.24). The expression and comprehension level were relatively good among all clients, meaning the participants generally or usually understood instructions given.

**Table 1:** Participant characteristics (*N* = 399) for the three types of organizations.

	**Gender**	**RES**		**DAY**		**ACT**	
**Number of participants**	**M**	**86**		**105**		**20**	
	**F**	**38**		**101**		**49**	
	**Gender**	**Mean**	**SD**	**Mean**	**SD**	**Mean**	**SD**
Age (y)	M	65.3	11.8	72.4	11.6	69.8	7.8
	F	78.4	10.3	74.6	10.8	72.4	8.4
BMI (kg/m^2^)	M	24.0	4.1	23.5	3.9	24.5	3.5
	F	23.6	4.9	24.6	4.4	25.8	4.7
ADLh (0–6)	All	0.85	1.06	0.67	0.88	0.00	0.00
CPS (0–6)	All	2.01	1.06	1.29	1.26	0.24	0.49
COMM (0–8)	All	1.95	1.86	1.22	1.53	0.27	0.83

*ADLh = activity of daily living hierarchy scale (0-6), CPS = cognitive performance scale (0-6), COMM = communication scale (0-8). RES = residential facilities group, DAY = day rehab/dementia day care/senior care centers group, ACT = senior activity/community centers group, sd = standard deviation*.

### Effectiveness of Strength Training

**Figure 2:**
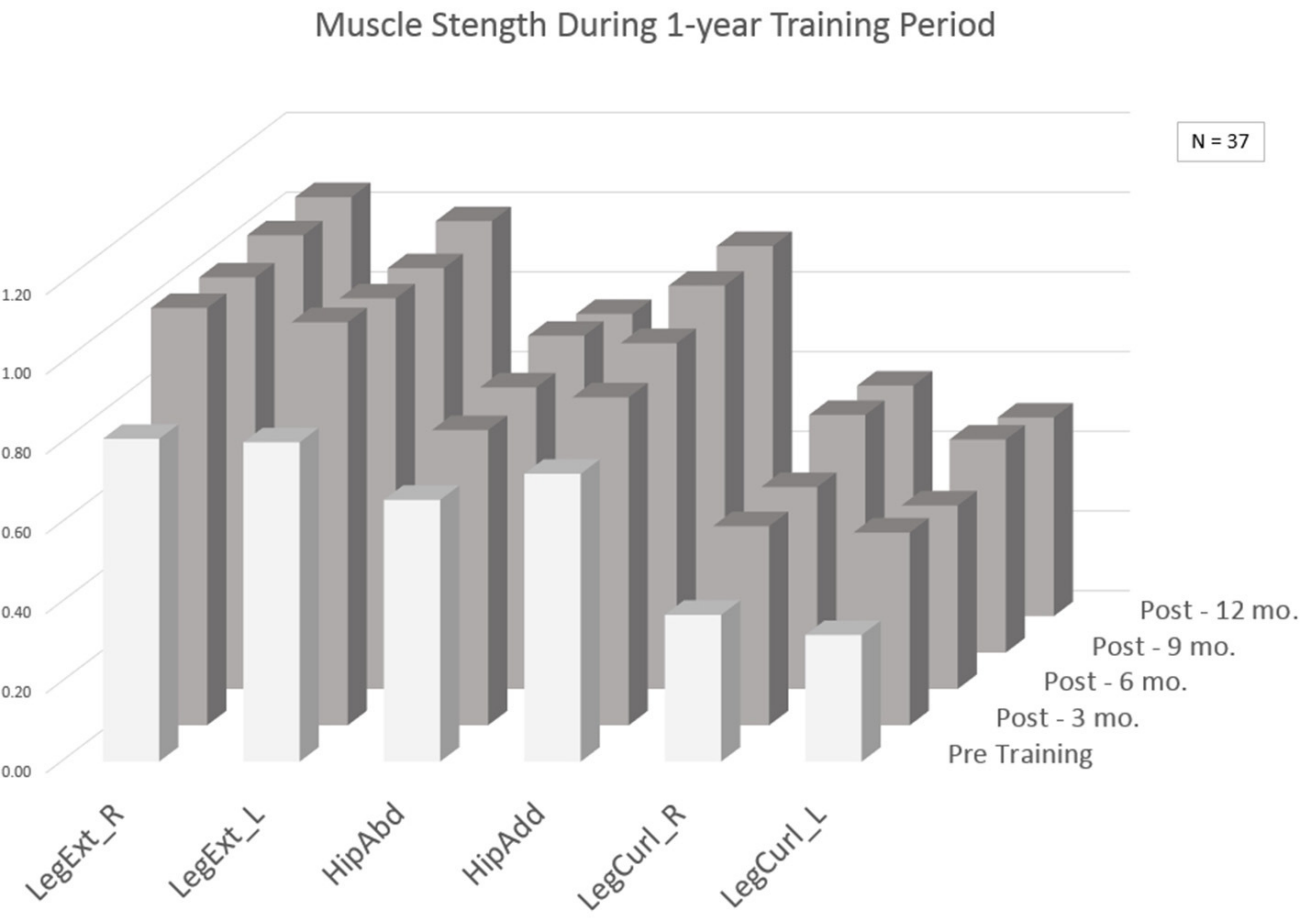
Average strength results (kgf/body weight) for a subgroup of 37 participants who trained for an extended period. LegExt_R, leg extension right leg; LegExt_L, leg extension left leg; HipAbd, hip abduction; HipAdd, hip adduction; LegCurl_R, leg curl right leg; LegCurl_L, leg curl left leg.

The effectiveness of training was analyzed from a before and after perspective comparing pre and post exercise assessments (*p* < 0.05). Table 2 presents the results comparing the pre and post tests for females and males separately. The average improvement rates (post/pre %) in the lower body isometric strength tests were in the range of 6.1–48.9% depending on test and gender. The highest improvement rates were found in LegCurl (ave 11.1–48.8%), LegExt (ave 10.2–24.0%) and the lowest in HipAbd/HipAdd (ave 7.0–15.8%). Some significant improvements were seen in functional tests such as FTSTS with an 11.4% improvement rate for ACT participants (females) and an 8.9% improvement rate for DAY participants (females). Walking speed improved by about 8.7% for male RES clients. In balance, improvements were mainly seen among female DAY participants based on the Berg Balance Scale with an average improvement rate of 9.6%. A subset of 37 participants who had trained actively for 1 year and were assessed quarterly (approx. at 3, 6, 9, 12 months), was analyzed (Figure 2). In this group, the largest improvements in strength were received from the first 3 months of training followed by a leveling off effect. For LegExt strength improved by 20% from the first 3 months of training, after which the improvements leveled off. Only for HipAdd, there was a continuous improvement during the entire 1-year follow-up in this subgroup.

**Table 2:** Pre-post changes for tests (*N* = 399).

		**RES**				**DAY**				**ACT**			
**Test**		**m1**	**m2**	**ch%**	***P***	**m1**	**m2**	**ch%**	***P***	**m1**	**m2**	**ch%**	***P***
LegExt Right (kgf/kg)	M	0.97	1.07	10.2	0.021	1.08	1.20	11.0	0.000	1.47	1.56	6.1	0.307
	F	0.67	0.79	18.6	0.004	0.76	0.87	13.7	0.000	0.87	1.02	16.3	0.000
LegExt Left (kgf/kg)	M	0.96	1.07	11.5	0.021	1.04	1.17	12.6	0.000	1.39	1.61	16.0	0.006
	F	0.68	0.79	17.4	0.002	0.76	0.87	14.6	0.000	0.82	1	21.3	0.000
LegCurl Right (kgf/kg)	M	0.43	0.52	20.8	0.001	0.52	0.57	11.1	0.001	0.69	0.87	24.0	0.009
	F	0.33	0.39	19.8	0.079	0.34	0.43	28.3	0.000	0.38	0.52	36.0	0.000
LegCurl Left (kgf/kg)	M	0.40	0.53	31.6	0.000	0.49	0.55	12.7	0.008	0.67	0.85	27.7	0.000
	F	0.31	0.40	32.4	0.013	0.33	0.42	25.6	0.000	0.34	0.51	48.8	0.000
HipAbd (kgf/kg)	M	0.73	0.82	12.9	0.000	0.77	0.81	5.1	0.011	1.1	1.16	6.3	0.073
	F	0.60	0.68	13.9	0.011	0.60	0.66	11.4	0.000	0.77	0.84	9.3	0.001
HipAdd (kgf/kg)	M	0.77	0.86	11.7	0.001	0.88	0.95	7.3	0.000	1.26	1.35	7.0	0.037
	F	0.68	0.72	6.0	0.117	0.62	0.72	15.8	0.000	0.76	0.86	13.9	0.000
FTSTS (sec)	M	15.04	14.60	−2.9	0.298	15.73	14.65	−6.9	0.094	12.39	10.43	−14.6	0.067
	F	15.49	13.29	−14.2	0.295	17.67	16.09	−8.9	0.019	13.42	11.89	−11.4	0.005
BBS Short (score 0–16)	M	9.92	10.82	9.0	0.006	8.62	9.45	9.7	0.005	13.11	13.11	0.0	1.000
	F	9.31	9.84	5.7	0.329	8.05	8.83	9.6	0.017	12.2	12.33	1.0	0.698
FAT (%)	M	22.77	22.17	−2.6	0.164	22.28	22.22	−0.2	0.764	22.91	23.28	2.4	0.365
	F	33.18	33.68	1.5	0.685	35.05	35.64	1.7	0.033	36.59	36.7	0.3	0.774
SPEEDN (m/s)	M	0.81	0.90	11.4	0.030	0.69	0.69	−0.8	0.753	1.02	1.01	−0.1	0.980
	F	0.57	0.56	−1.7	0.697	0.60	0.65	8.5	0.061	0.89	0.94	5.2	0.197
SPEEDF (m/s)	M	1.11	1.21	8.7	0.024	0.95	0.97	1.8	0.576	1.56	1.6	2.7	0.679
	F	0.77	0.76	−0.8	0.869	0.83	0.86	3.3	0.263	1.29	1.33	2.8	0.367

LegExt Right, Leg Extension Right; HipAbd, Hip Abduction; HipAdd, Hip Adduction; FTSTS, five times sit to stand; BBS, Berg Balance Scale (sum of last four items); FAT, total body fat %; SPEEDN, normal walking speed; SPEEDF, fast walking speed; RES, residential facilities group; DAY, day rehab/dementia day care/senior care centers group; ACT, senior activity/community centers group. P-value (p) calculated with the paired t-test for the difference between pre (m1) and post-test (m2). Change expressed as percent change (ch); that is, 100^*^(m2-m1)/m1. Gray are those values significant at p < 0.05 level.

**Figure 3:**
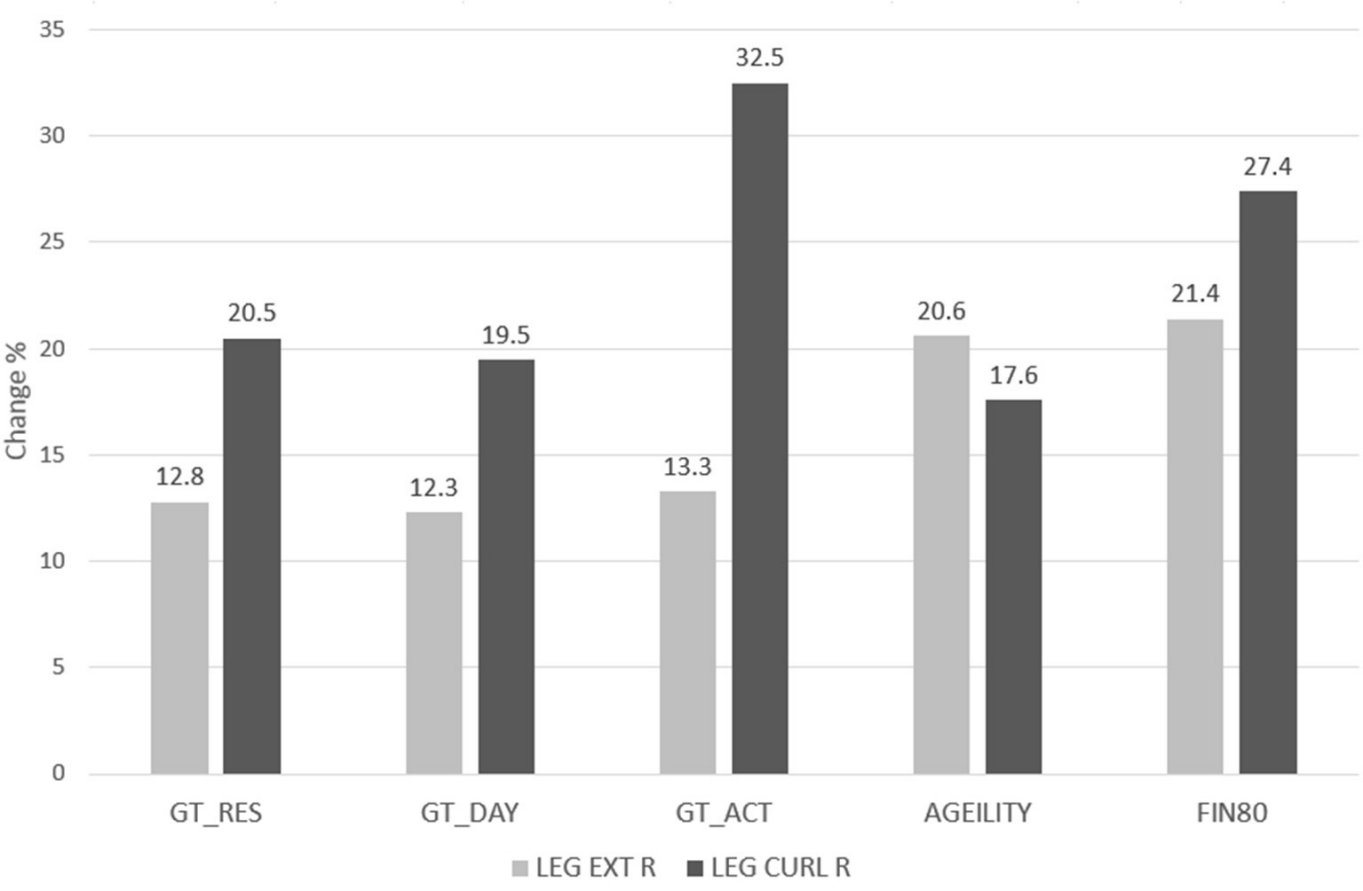
Post-pre change in terms of improvement rate calculated as mean (post-pre)/mean (pre) for Right Leg Extension and Curl strength for Gym Tonic (*N* = 399) and “reference” groups and averaged over gender. AGEILITY (*N* = 12) and FIN80 (*N* = 28) were tightly supervised exercise groups.

For comparison of outcomes, we used two smaller datasets, one from Singapore and one from Finland, where we had used identical assessment methods and gym technology for training with elderly (Figure 3). These groups had a tighter control in the intervention but otherwise applying the same exercise methods as Gym Tonic. Ageility refers to a Singaporean exercise group performed in the Gym Tonic demonstration gym operated by PulseSync (7M + 5F, 72 ± 6 y, 12 weeks of supervised strength training). FIN80 refers to an exercise group of Finnish community dwelling 80-year olds (9M+19F, 10 weeks of training). The results of the Gym Tonic sites in terms of improvement rates were comparable to these reference groups. Based on the interRAI assessments, some indications of clients becoming more independent functioning could also be detected: ADLh from 0.51 (pre) to 0.42 (post), CPS from 1.32 (pre) to 1.28 (post), and COMM from 1.27 (pre) to 1.17 (post).

### Progressiveness of Strength Training

Individual exercise data was obtained from each training machine, providing the opportunity to analyze the progressiveness of the resistance training during the intervention period. The starting training load was calculated as the average for the first 2 weeks of training, and the ending load as the average of the final 2 weeks of the 90 days training period. The data indicated that the training was progressive with the load increment at an average of 21% toward the end of the intervention. Compared to the reference groups the progressiveness was somewhat lower. The average increase in training loads was 36% (F) and 57% (M) for the Ageility group and about 58% for FIN80 group.

### Staff Responses

The implementation process was evaluated using surveys. The survey studies were done about 3 years after the start of the implementation giving the respondents enough time and perspective to evaluate the implementation. Survey #1 was sent to those who had participated in the training in Finland (Group 1), and a total of 40 out of 49 answered (response rate 82%). Survey #2 was posted to those actively working with Gym Tonic, but not trained in Finland (Group 2), and a total of 22 out of 41 answered the survey (response rate 54%). At the time of answering the questionnaire, 70–80% of the respondents worked full time or part time with Gym Tonic.

**Figure 4:**
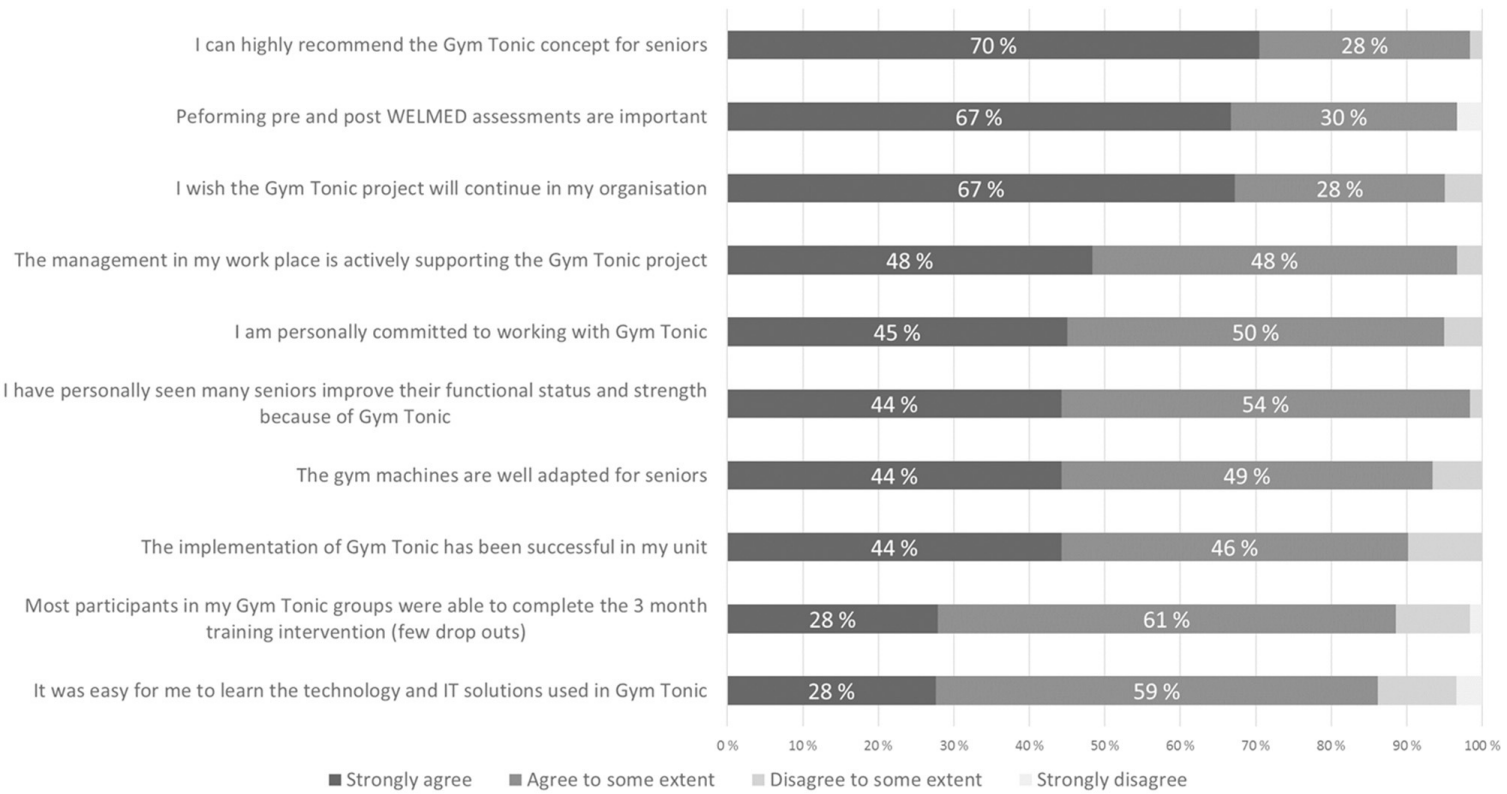
Implementation questionnaire. Staff responses of those trained in Finland for Gym Tonic (*N* = 40) and those trained locally for Gym Tonic (*N* = 22).

The results from the two surveys were similar, we have therefore pooled them (Figure 4). To the statement “I can highly recommend the Gym Tonic concept to seniors” 70% strongly agreed and 28% agreed to some extent. The respondents also felt the management had been supportive of the project. To the statement “The implementation of Gym Tonic has been successful in my unit” 44% strongly agreed and 46% agreed to some extent. To the statement “Performing the Welmed assessments were important” 67% strongly agreed and 30% agreed to some extent. Most of the respondents also wished the project would continue. Of the respondents over 44% strongly agreed and 49% agreed to some extent that the gym machines were well-adapted for elderly. Good compliance was generally seen in terms of few dropouts and the frontline staff had seen many improve their functional status and strength.

On the open ended questions what had contributed to the success of the implementation the following factors were emphasized: strong sponsorship by foundation, good support by management, user friendly system, motivated trainers, seeing elderly improve after post-tests, good location of Gym Tonic sites (near persons' homes), good support from vendors, safe machines, dedicated staff and volunteers, proper training and good planning. On the question what had contributed to the lack of success the following causes were mentioned: lack of manpower, lack of trained staff/physiotherapists, finding suitable pool of participants, lengthy assessments, lack of supervision/follow-up, restrictions from supervising levels, lack of understanding of concept in the business sense, and lack of management's understanding of the program.

## Discussion

This study has provided a comprehensive overview of implementing strength training in different elder care settings. Several lessons can be learned from the PBE viewpoint. The most important lesson is that strength training can be implemented successfully in real settings producing effective and repeatable outcomes. The study data showed significant improvements in muscle strength in each of the three settings including those living in the community and those residing in facilities. In terms of outcomes, the improvement rates in muscle strength were comparable to those reported in meta-analysis of research studies by for example, Lopez et.al., observing increases of 6.6–37% in maximal strength of strength training and multimodal training (40), and Peterson reporting an average of 33% increase in muscle strength on resistance training for muscular strength in older adults (10). The results of Gym Tonic also corresponded to reference groups using identical technology and progressive training.

The data driven approach provided relevant information for understanding the implementation process. Several practice patterns could be drawn from the data. The first 3 months of resistance training produced the largest improvements in muscle strength. This suggests that a relatively short exercise period can be used for improving physical strength, where after the challenge is to maintain it. This finding is based on a relativiely small data set (*n* = 37) and a pattern found for those in the residential group. The progressiveness of the resistance training succeeded to a degree, showing the users were able to apply the gym technology appropriately. In the progressiveness of training, some cautiousness was detected in raising the training resistance, which is to be expected in the introduction of new technology. Increased progressiveness and higher starting loads could potentially produce better outcomes. The data indicated no clear dose response relation between training load and improvement in strength. Therefore, the improvements in muscle strength were most likely due to improvements in neural activation (27) and/or improved muscular metabolic capacity (11). Longer training periods are generally needed for building muscle mass. In terms of changes in functional dependency, the post-tests of the interRAI assessments pointed in the right direction, but more data is needed to draw conclusions from the perspective of improving independency. The sites targeted relatively young persons and those who were physically relatively independent. Independent in ADLs doesn't necessarily mean that the person is in good physical condition and not in the need of training, as shown in this study.

The surveys suggested that the implementation had been successful from the view of frontline staff. The adoption of new technology succeeded without major hurdles. Both therapists and specialists could be trained with a relatively short training course in the use of different technologies and applying strength training for elderly. The sites were able to independently plan and carry out the proposed interventions based on the training given. This is encouraging as training is usually a major component of implementing new concepts. The main challenge in the training was the cultural change of putting elderly persons in gyms. These issues had to be covered relatively extensively in the transition from a physically passive fall prevention to physically active fall prevention. From a policy perspective, the Senior Activity Centers offers an interesting model for reaching a large number of elderly in the community. In a densely populated city like Singapore such centers can be set up conveniently. Finally we may point out that the comprehensive assessment approach adopted here is in line with the philosophy behind the concept of intrinsic capacity (IC) as promoted by WHO (22). The focus is shifted from symptoms to the mental and physical capacities of the person, which are important for healthy aging. One limitation of the study though is the lack of participant perspective which must be remedied in future studies.

## Conclusion

The Gym Tonic project offers important insights and directions on how to implement progressive strength training on a large scale across different elder care settings. The following conclusions can be drawn from the study:

Positive outccomes:

- The approach yielded consistent improvement rates in muscle strength comparable to results from randomized clinical trials (meta-analysis studies) showing that effective outcomes can be achieved in real life environments.
- Significant improvement rates in muscle strength were found in all three types of sites demonstrating the vast potential of strength training to promote resilient aging.
- The data supported a 3-month training intervention as an effective way of introducing strength training, this could be useful for policy makers looking for practical solutions in the war against frailty.
- Frontline staff were successfully trained in the use of the technology for gym training and assessments, suggesting that technology solutions can be applied in real life practice for strength training.
- Practice patterns could be detected using the data-driven approach highlighting the need and capabilities of information and assessment systems for decision making and continuous quality improvement.

Lessons learned:

- Although frontline staff underwent structured training, some of them may not adhered fully to our assessment and training protocol due to manpower and other factors. To improve this, the project introduced regular refresher courses and also frequent on-site visits to improve the overall compliance.
- Initially most organizations were using physiotherapists to run Gym Tonic, thus limiting the scalability of the programme given the high manpower cost and scarcity of resources. Many of them switched to hiring exercise therapists or wellness coaches, while leaving the physiotherapists to handle the more complex cases, for example elderly who are very frail and/or with medical conditions (especially those in the nursing homes).
- While participants were briefed on their pre-assessment and post-assessment results prior and on completion of the intervention, some may not be able to comprehend and relate the results to their ADLs. Helping the trainers to explain and better relate the results this were improved and included in the training.
- Getting community-dwelling frail elderly to participate is logistically challenging as transport and sometimes, caregiver may be needed as many of them need at least supervisory assistance. In addition, the time needed for them to complete the exercise is typically longer. To address this, most sites are now using the afternoon non-busy/quiet period (from 1–3 pm) to handle such elderly so as to give them more quality time and supervision.

As the data grows more lessons can be drawn for introducing strength training for elderly. The greatest challenges do not seem to be technological, but rather changing of mindsets and implementing concepts that work. This study was done to increase that knowledge. More practice-based evidence is needed for the industry, policy makers, and for an aging population to motivate people to use exercise as a key remedy and medicine for resilient aging.

## Data Availability Statement

The datasets generated for this study will not be made publicly available The researchers were given access to the data for analysis, but not make it publicly available.

## Ethics Statement

Ethical review and approval was not required for the study on human participants in accordance with the local legislation and institutional requirements. Written informed consent for participation was not required for this study in accordance with the national legislation and the institutional requirements.

## Author Contributions

MB and FB analyzed the data and wrote the paper. KT, GL, and LN have read and commented on the manuscript. All authors contributed to the article and approved the submitted version.

## Conflict of Interest

MB is a co-founder of RaiSoft. KT is a founder of Pulse Sync. The remaining authors declare that the research was conducted in the absence of any commercial or financial relationships that could be construed as a potential conflict of interest.

## Publisher's Note

All claims expressed in this article are solely those of the authors and do not necessarily represent those of their affiliated organizations, or those of the publisher, the editors and the reviewers. Any product that may be evaluated in this article, or claim that may be made by its manufacturer, is not guaranteed or endorsed by the publisher.
